# Complete genome sequence analysis of plant growth-promoting bacterium, *Isoptericola* sp. AK164 isolated from the rhizosphere of *Avicennia marina* growing at the Red Sea coast

**DOI:** 10.1007/s00203-023-03654-1

**Published:** 2023-08-14

**Authors:** Amal Khalaf Alghamdi, Sabiha Parween, Heribert Hirt, Maged M. Saad

**Affiliations:** DARWIN21, Center for Desert Agriculture (CDA), Biological and Environmental Science and Engineering Division (BESE), King Abdullah University of Science and Technology (KAUST), 23955-6900 Thuwal, Saudi Arabia

**Keywords:** *Isoptericola*, Genome, PGPR, Red Sea, Mangrove, *Arabidopsis thaliana*

## Abstract

*Isoptericola* sp. AK164 is a Gram-positive, aerobic bacterial genus from the family Promicromonosporaceae, isolated from the root rhizosphere of *Avicennia marina*. AK164 significantly enhanced the growth of the *Arabidopsis thaliana* plant under normal and saline conditions. These bacteria can produce ACC deaminase and several enzymes playing a role in carbohydrate hydrolyses, such as cellulose, hemicellulose, and chitin degradation, which may contribute to plant growth, salt tolerance, and stress elevation. The genome sequence AK164 has a single circular chromosome of approximately 3.57 Mbp with a GC content of 73.53%. A whole genome sequence comparison of AK164 with type strains from the same genus, using digital DNA–DNA hybridization and average nucleotide identity calculations, revealed that AK164 might potentially belong to a new species of *Isoptericola*. Genome data and biochemical analyses indicate that AK164 could be a potential biostimulant for improving agriculture in submerged saline land.

**Supplementary Information:**

The online version contains supplementary material available at 10.1007/s00203-023-03654-1.

## Introduction

The coastal areas of the Red Sea are one of the unique extreme environments with high biodiversity revealing large microbial communities with a high abundance of Actinobacteria in the mangrove forests of *Avicennia marina* (Alzubaidy et al. 2016)*.* Those Actinobacteria are Gram-positive bacteria that show a remarkable range of morphologies, including unicellular cocci or rods and differentiated branched multicellular bacteria (van Bergeijk et al. 2020). They usually grow in numerous habitats, especially extreme environments, such as salt marshes, hot springs, alkaline saline soils, deep-sea sediments, and the microbiome of a higher eukaryote (Qin et al. 2016). The ability of the Actinobacteria to adapt to those extreme environments is correlated to their production of bioactive natural products and secondary metabolites with diverse functions, including the cycling of complex carbon substrates in benthic ocean habitats (Mincer et al. 2002). On the other hand, Actinobacteria are important plant symbionts that play a crucial role in enhancing plant growth as plant growth promotion bacteria (PGPB) and protection against pathogens (Eida et al. 2018). The genus *Isoptericola* is found to be involved in phosphate uptake and the degradation of cellulose, hemicellulose, and chitin (Su et al. 2021). Noteworthy, it has been revealed that ACC deaminase-producing strains of *Isoptericola* stimulated the growth of the host plant and influenced flavonoid accumulation, which is known for prominent roles in stress alleviation (Qin et al. 2014). There is a need to study this phylum of bacteria as bioactive compound producers along with the metabolism and genetic structure governing this ecological context. Here, we report the genome sequence analysis of *Isoptericola* sp. AK164, plant growth promoting rhizosphere bacterium isolated from the root rhizosphere of *Avicennia marina* growing at the Red Sea shore in Thuwal, Saudi Arabia, highlighting their plant growth properties and potential biotechnological application. Genome sequencing and analysis, of rhizosphere bacteria e.g. AK164 for their potential plant growth promoting activities under different growth conditions will speed up the applications of biostimulants in smart agriculture system as an eco-friendly solution to mitigate the negative impact of climate change.

## Material and methods

### Isolation and growth conditions of AK164

AK164 was isolated from the rhizosphere of the intertidal plant *A. marina* growing in the coastal area of the Red Sea (GPS: 22.339914° N, 39.087972° E), Thuwal, Saudi Arabia on Reasoner's 2A agar (R2A) medium (Merck, Germany) + 0.5 M NaCl. The pure culture of AK164 was regularly growing in Zobell 2216E agar (ZM) (Bio Basic Asia Pacific Ltd, Singapore) at 30 °C.

### Biochemical assays

Plant growth-promoting (PGP) traits were evaluated by using clearing assays. The ability of AK164 to solubilize phosphate was assessed on Pikovskaya’s (PVK) agar plates (M520, Himedia). Using Blue Agar CAS assay determined siderophores' production as described by Louden et al. (2011), Louden et al. (2011). The IAA production was tested according to Patten and Glick (2002). The AK164 strain was isolated from the coastal environment at the Red Sea with relatively high temperatures reaching > 45 °C with hypersaline conditions in which NaCl was the main salt. To use these microorganisms as plant growth promoters, we assessed their ability of AK164 to grow at moderately high temperatures (37 and 45 °C), and saline media (0–5 M NaCl) were assessed using Zobell marine agar (ZM) broth. Our data as well as previous data reported in the Red Sea coastal area showed that salinity reaches 35 PSU and high electrical conductivity of (39 uS/c) indicating higher dissolved sodium chloride in the soil (Alhassan and Aljahdali 2021).

### Arabidopsis salt stress tolerance assays

*Arabidopsis thaliana* Col-0 seeds were surface sterilized for 10 min in 70% ethanol (v/v) solution supplemented with 0.05% triton-X, then washed 4 times with 100% ethanol. The seeds were left to dry in aseptic conditions on sterilized filter paper until use. Seed colonization with bacteria and plant growth conditions was conducted as described previously (Andres-Barrao et al. 2017). For the inoculation procedure, 100 µl of the bacterial culture was used with 10E8 cells, and plant growth was monitored after 15 days. The total fresh weight (FW) of whole seedlings was recorded to evaluate the effect of the bacterial treatment on plant growth under normal and salt conditions. The data from the plant screening assay were subjected to non-parametric one-way ANOVA or the Kruskal–Wallis test (Kruskal and Wallis 1952). The statistical difference is based on Paired t-test/Dunn's multiple comparisons tests (****P < 0.0001). All statistical analysis was done using GraphPad Prism version 9.5.0 (525) software (https://graphpad.com).

### Genomic DNA extraction

Total genomic DNA was extracted from the pure cultures of AK164, using the GenElute™ Bacterial Genomic DNA Kit (Sigma Aldrich, Germany) according to the manufacturer’s instructions. DNA integrity, quality, and quantity were assessed by using the agarose gel electrophoresis 1%, NanoDrop 2000 spectrophotometer (Thermo Fisher Scientific, Schwerte, Germany), and the concentration by Qubit dsDNA high-sensitivity (HS) Kit (Thermo-Fischer Scientific).

### Whole genome sequencing and function annotation

Genomic sequencing and assembly were carried out at Novogene Bioinformatics Technology Co., Ltd. (Singapore). Single-molecule real-time (SMRT®) sequencing was performed on PacBio Sequel II/IIe systems. FALCON software (falcon-kit = 1.8.1) was used for the whole genome assembly (Chin et al. 2016). It follows the design of the previously developed Hierarchical Genome Assembly Process (HGAP), using greatly optimized components. Polishing and circularization of assembled genome done by Arrow (2.3.3) and circulator (1.5.5), respectively (Page et al. 2016). BUSCO (4.0.2) (Benchmarking Universal Single-Copy Orthologs, https://busco.ezlab.org) quantitative measurements were used to assess the genome assembly. Genome annotation was done by Novogene using their in-house pipeline. It covers coding genes, repetitive sequences, and non-coding RNA. For repeat annotation, the interspersed repetitive sequences were predicted using the RepeatMasker (http://www.repeatmasker.org/). The tandem Repeats were analyzed by the TRF (Tandem repeats finder). For ncRNA annotation, transfer RNA (tRNA) genes were predicted by the tRNAscan-SE (Blin et al. 2021). Ribosome RNA (rRNA) genes were analyzed using the RNAmmer (Lagesen et al. 2007). Small nuclear RNAs (snRNA) were predicted by BLAST against the Rfamdatabase. Augustus (http://bioinf.uni-greifswald.de/augustus/) and GeneWise (http://www.ebi.ac.uk/~birney/wise2/) software for coding gene prediction have been used. The functional annotations were performed using several databases such as respective GO (Gene Ontology) (Galperin et al. 2021), KEGG (Kyoto Encyclopedia of Genes and Genomes) (Kanehisa et al. 2004), KOG (EuKaryotic Orthologous Groups) (Galperin et al. 2021), NR (Non-Redundant Protein Database) (Brown et al. 2015), Swiss-Prot, and TrEMBL (Bairoch and Apweiler 2000). Identification of secondary metabolite encoding gene clusters was performed using antiSMASH v.4.2.0. (Blin et al. 2021).

### Phylogenomic classification of AK164

For a whole genome-based taxonomic analysis, the genome sequence data were uploaded to the Type (Strain) Genome Server (TYGS), (https://tygs.dsmz.de). All pairwise comparisons among the set of closely related genomes were done using the Genome BLAST Distance Phylogeny (GBDP) (Lagesen et al. 2007; Camacho et al. 2009) approach under the “coverage” algorithm and distance formula d5. These distances were finally used to determine the 14 closest strain genomes to AK164. Digital DDH values and confidence intervals were calculated using the recommended settings of the GGDC 3.0. The resulting intergenomic distances were used to infer a balanced minimum evolution tree with branch support via FASTME 2.1.6.1 including SPR post processing (Lefort et al. 2015). Branch support was inferred from 100 pseudo-bootstrap replicates each. The trees were rooted at the midpoint and visualized with PhyD3 (Kreft et al. 2017). Furthermore, Orthologous Average Nucleotide Identity Tool (OAT) software (Lee et al. 2016) was used to calculate the OrthoANI values between the close strains belonging to the AK164. The average amino acid identity of AK164 with all its closely related species were performed using the EzAAI program (Kim etal.2021) which uses MMSeqs2 to calculate the AAI across the CDS profile of the genome.The phylogenetic tree was constructed using its cluster package.

### Data deposition

The genome sequence of AK164 was deposited in NCBI/DDBJ/EMBL database under the accession number CP119106 with BioProject no. PRJNA929179.

## Results and discussion

**Fig. 1:**
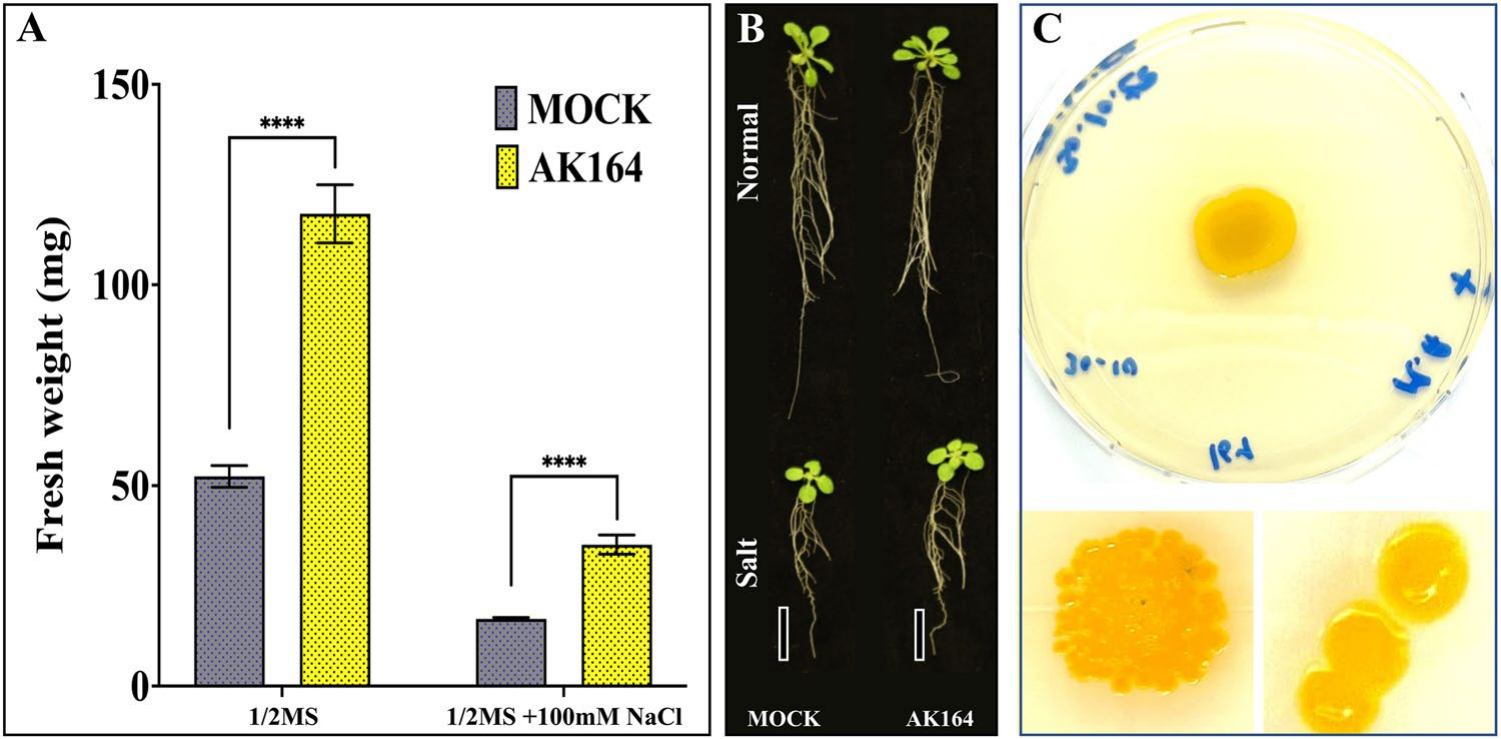
*Isoptericola* sp. AK164 general features and characteristics. **A** Bar plots showing means of fresh weight (mg) of *Arabidopsis thaliana* grown on 1/2MS and inoculated with AK164 compared with non-inoculated (mock) plants. **B** Growth of 20 days old *A. thaliana* in both normal and salt stress conditions, either MOCK plants or inoculated with AK164, scale bar = 2 cm. **C** The colony morphology of AK164 *Isoptericola* sp. AK164 on ZM agar. All plots represent the mean of three biological replicates (n = 24). Error bars represent SE. Asterisks indicate a statistical difference (**** *P* < 0.0001)

*Isoptericola* sp. AK164 is Gram-positive, cocci-shaped, non-motile with 300–400 nm diameter, with characteristic circular and bright yellow colonies, observed when grown on Zobell marine Agar at 30 °C (Fig. 1C). AK164 tolerates heat and salinity stress and grows in temperatures up to 45 °C and in/on up to 5 M NaCl-supplemented LB, which displays salt (NaCl) and high-temperature tolerance (Supplement Table S1). The ability of AK164 to tolerate salt stress contributes to the survival of AK164 in the coastal intertidal zones of mangroves (*A. marina*) and marine environments. The qualitative evaluation of PGP traits showed that AK164 can produce siderophores and IAA (Supplement Table S1), giving AK164 a potential application as PGPR. *In planta*, AK164 caused enhancement of *A. thaliana* growth under both normal (½ MS and salt stress conditions of 100 mM NaCl; beneficial increase > 110% compared with the non-colonized control plants). After 12 days of growth, *A. thaliana* seedlings treated with AK164 showed bigger shoot and root systems (Fig. 1B) and an increment of 50% in fresh weight (Fig. 1A). This growth promotion could be due to IAA production or 1-aminocyclopropane-1-carboxylate (ACC) deaminase or other metabolic compound synthesis by AK164.

**Fig. 2:**
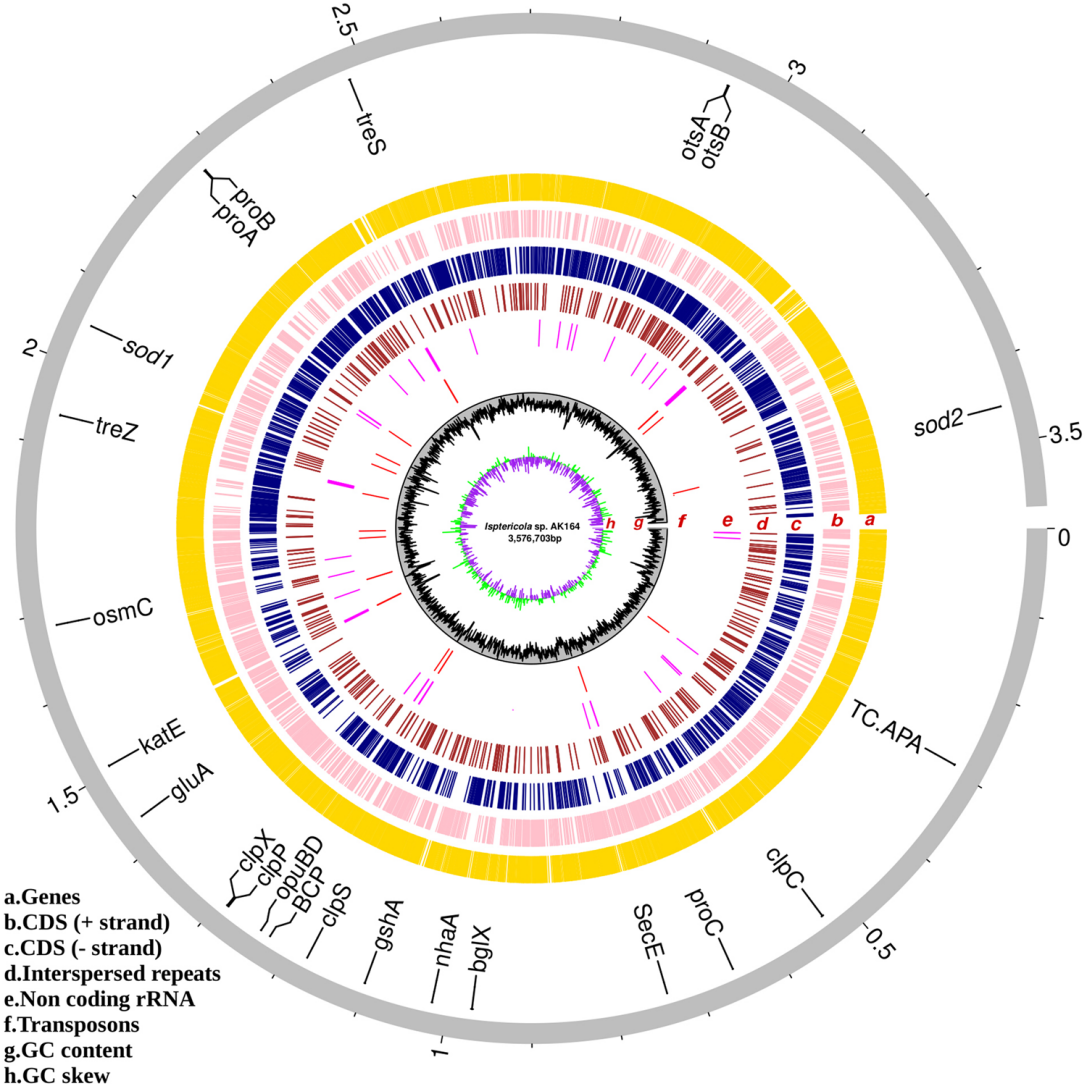
Genome map of *Isoptericola* sp. AK164. The whole genome sequence size is split in Mbs and shows the specific key regulatory genes and essential genomic features. From the outer to the inner circle, representation is as follows: **a** genes (gold); **b** forward strand coding sequences (pink); **c** reverse strand coding sequences (navy); **d** Tandem repeats (brown); **e** noncoding rRNA(magenta); **f** Interspersed repeats (red); **g** %GC content (black) [Supplement Table S5, B]; h. GC skew (green and purple correspond to a higher and lower value, respectively [ Supplement Table S5, A]) *treZ* maltooligosyltrehalose trehalohydrolase [EC:3.2.1.141], *treS*: maltose alpha-D-glucosyltransferase/alpha-amylase [EC:5.4.99.16 3.2.1.1], *ots*A: trehalose 6-phosphate synthase [EC:2.4.1.15 2.4.1.347], *ots*B trehalose 6-phosphate phosphatase [EC:3.1.3.12], *pro*C pyrroline-5-carboxylate reductase [EC:1.5.1.2], *pro*A glutamate-5-semialdehyde dehydrogenase [EC:1.2.1.41], *pro*B glutamate 5-kinase [EC:2.7.2.11], *TC.APA* basic amino acid/polyamine antiporter, APA family, *nhaA* Na + :H + antiporter, NhaA family, *gshA* glutamate–cysteine ligase [EC:6.3.2.2], *sod*1 superoxide dismutase, Cu–Zn family [EC:1.15.1.1], *sod2* superoxide dismutase, Fe–Mn family [EC:1.15.1.1], *ka*tE catalase [EC:1.11.1.6], *clp*X ATP-dependent Clp protease ATP-binding subunit ClpX, *clp*P ATP-dependent Clp protease, protease subunit [EC:3.4.21.92], *clp*S ATP-dependent Clp protease adaptor protein, *clp*C ATP-dependent Clp protease ATP-binding subunit, *BCP* peroxiredoxin Q/BCP [EC:1.11.1.15], *osm*C lipoyl-dependent peroxidase [EC:1.11.1.28], *opuB*D osmoprotectant transport system permease protein, *gluA* glutamate transport system ATP-binding protein [EC:7.4.2.1], *bgl*X beta-glucosidase [EC:3.2.1.21], *sec*E Preprotein translocase subunit

Sequencing of the AK164 strain using PacBio technology resulted in 14,559 reads with a mean read length of 73,030 bp and estimated genome coverage of 297X. The assembled genome consists of one contig of 35,767,03 bp with a GC content of 73% (Supplement Tables S5A, B). In total, 3211 coding genes have been annotated in the AK164 genome, covering 91.44% of the genome. RNA-noncoding genes, including 25 tRNAs and 9 rRNAs were predicted in the genome (Fig. 2, Table 1). Special emphasis was made on capturing the repeat regions in the genome as they play important roles in genome evolution and adaptation to environments. We identified mainly two kinds of repeats in the genome, Tandem (22) and Interspersed (1094) (Fig. 2, Table 1).

**Table 1:** Summary of *Isoptericola* sp. AK164 genome features

Feature	Chromosome
Genome Size (bp)	3,576,703
GC content (%)	73.54
Gene Number	3211
% of Genome (Genes)	91.44
CDS	3211
16S-23S-5S operons	3-3-3
tRNAs	49
long terminal repeat LTR	13
DNA	5
Tandem repeats TRs	566
Minisatellite DNA	521
Microsatellite DNA	7

**Fig. 3:**
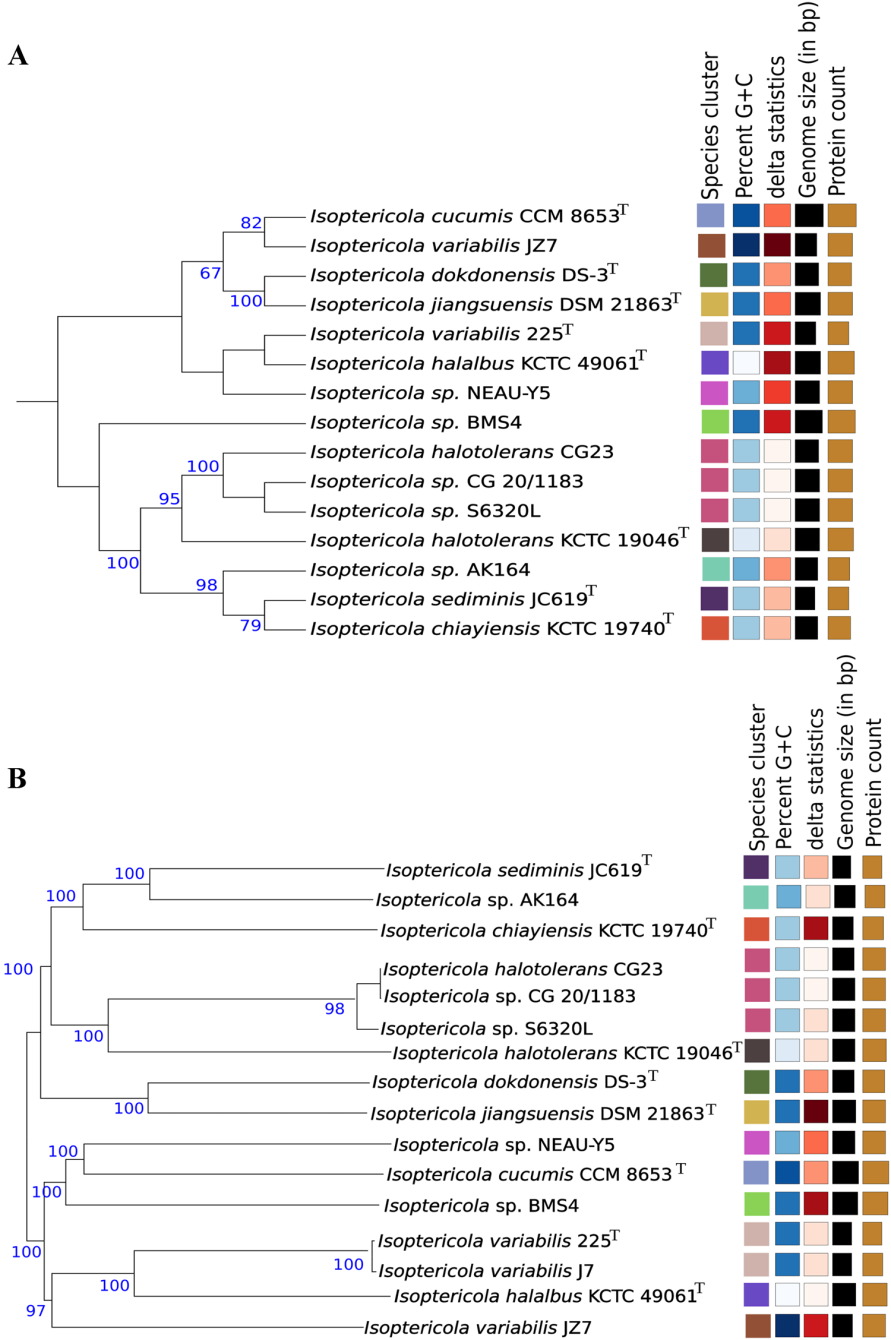
Phylogenomic classification of *Isopetricola* sp. AK164 Tree inferred with FastME 2.1.6.1 (Lefort et al. 2015) from GBDP distances. The branch lengths are scaled in the GBDP distance formula *d5* and rooted at the mid-point. The numbers above branches are GBDP pseudo-bootstrap support values > 60% from 100 replications. **A** Tree calculated from 16S rDNA gene sequences with an average branch support of 70.8%. B Whole genome sequences-based tree with an average branch support of 96.3%. Type strains are indicated by ^T^

To determine the accurate taxonomic position of AK164, 16S and whole-genome-based taxonomic analysis was undertaken with the Type Strain Genome Server (TYGS) platform (Page et al. 2016) as shown in Fig. 3A, B. The TYGS results show that AK164 is most closely related to *Isoptericola sediminis* JC619 with dDDH (d0) of 61.8% corresponding to high average branch support for the generated tree and high phylogenetic accuracy. However, to check the reliability of evolutionary distance assessment between bacterial species based on digital whole genome comparison, average nucleotide identity (ANI) was also measured. Complete genomes of *Isopetricola* species closely related to AK164 were retrieved from the NCBI GenBank database and subjected to Orthologous Average Nucleotide Identity calculation. As shown (Supplement Table S2 A, B) the highest OrthoANI value of 86.6% was obtained with *Isoptericola sediminis* JC619. However, the ANIo and dDDH values do not fit the species cut-off of 95% and 70%, respectively. Based on these results, AK164 may present a new species. In order to confirm the ANI, we calculated the average amino acids (AAI) using the EzAAI java program (Kim et al. 2021) but still none of the identities matches 100% with any of AK164 close clades which confirm that it is a potential new species (supplement Table S3, (Supplement Fig. S1).

Function analysis on AK164 was conducted using different platforms (see Materials & Methods); by using BlastKOALA, we identified 1702 genes (53%) with assigned functions (Supplement Fig. S2), while using KEGG gave 3042 genes (95%), COG 2570 genes (80%), GO 2290 genes (71%) (Supplement Figs. S3, S4) and Pfam 2290 genes (71%). The AK164 chromosome contained nine 16S-23S-5S rRNA operons and 49 tRNAs (Table 1). Genome mining revealed the presence of genes involved in environmental stress tolerance and responses, e.g., trehalose, proline, choline, and betaine (glycine-betaine), important osmoprotectants produced under saline stress. Complete glutamate and proline biosynthesis pathways (Supplement Fig. S5: Supplement Tables S6, S7) were identified and might be responsible for conferring salt tolerance. The genome of AK164 has a wide variety of enzymes and regulators that help bacteria to cope with oxidative stress, including superoxide dismutase (*sod*, AK164_GM001878; AK164_GM003108), catalase (*katE*, AK164_GM001364), peroxidase (AK164_GM002005) and two osmotically inducible proteins (*osmC*, AK164_GM001522, AK164_GM002579). These genes could explain the survival phenotypes of AK164 under high salt concentration (up to 5 M NaCl), and heat stress (up to 45 °C). In the genome of AK164, we have identified several enzymes responsible for the degradation of plant cell walls and their constituents, such as xylan and cellulose. These enzymes play a significant role in the bacterial internalization process within plant roots. Furthermore, our analysis revealed the presence of specific genes *uxaAC* and *uxuB* that are involved in the catabolism of D-galacturonate, a prominent monomer found in pectin (Supplement Table S4). These findings highlight the metabolic potential of AK164 and indicate its ability to utilize and break down key components of plant cell walls. This knowledge provides valuable insights into the potential interactions between AK164 and plants, particularly in the context of root colonization and endophytic lifestyles. Interestingly, all the genes required for flagella assembly were absent, confirming the non-motile nature of AK164.

The genome of AK164 revealed several genes involved in PGPR activity, e.g., AK164_GM000253 encodes for 1-aminocyclopropane-1-carboxylate (ACC) deaminase. ACC deaminase is involved in the metabolism of the immediate precursor of ethylene in ethylene biosynthesis and is one of the well-known PGP traits (Shen et al. 2013). Several siderophores were identified in AK164, e.g., Enterochelin. Bacteria produce siderophores to scavenge iron from the extracellular space and use specific transporters to recover the siderophore–iron complex, ensuring their iron supply. AK164 has a large inventory of genes (122 genes) encoding ABC transporters, including mineral inorganic and metal ion transporters, cobalamin/Fe^3+^-siderophore transport, Fe^3+^-hydroxamate, Fe^3+^-siderophore, Mn^2+^/Zn^2+^ transport, nitrate/sulfonate/bicarbonate transport, phosphate transport and Oligosaccharide and amino acid transport, e.g., cellulase, chitobiose, glucose (Supplement Table S4). Many ABC transporters could be linked to the capacity of AK164 to survive in different ecological niches (Andres-Barrao et al. 2021). Along with the uptake and exchange of nutrients, bacteria require various protein secretion systems for growth and interaction with plants. AK164 harbors a general secretion (*Sec*) and a twin-arginine translation (Tat) secretion pathway along with several genes encoding for a type 2-secretion system (T2SS). Those pathways are the most commonly used secretion systems to transport proteins across the plasma membrane (Natale et al. 2008).

AntiSMASH analysis revealed the presence of six clusters for secondary metabolite biosynthesis, two of which were identified as terpene with high similarity to carotenoid biosynthetic gene cluster BGC0000644 from *Dietzia* sp. CQ4 and BGC0000636 from *Brevibacterium linens*. NRPS-independent-siderophore with high similarity with FW0622 BGC0002690 biosynthetic gene cluster from *Verrucosispora* sp. FIM060022 (Zhao et al. 2020). And Ectoine (1,4,5,6-tetrahydro-2-methyl-4-pyrimidinecarboxylic acid) with a 70% similarity of BGC0000853: ectoine biosynthetic gene cluster from *Streptomyces* sp. The genes encoding for ectoine biosynthesis AK164_GM002457_*ect*A, AK164_GM002457_*ect*B, AK164_GM002457_*ect*C and its conversion to hydroxyectoine (AK164_GM001449_ *ect*D) were identified in AK164, and their presence could contribute to the osmotic and salt stress tolerance of AK164. Ectoine is an osmoprotectant agent found in several microorganisms and has a wide practical application in industries, including for skin protection and a potential medicine (Hermann et al. 2020). In addition, a cluster of Lasso peptide was found, Lasso peptide is a novel class of bacteria-derived ribosomal assembled and post-translationally modified peptides; they are found throughout the bacterial domain and exhibit a versatile array of biological functions (Hegemann et al. 2015).

## Conclusion

The results of the taxonomic analysis supports the notion that AK164 represents a novel species of *Isoptericola*, and further investigation is needed to fully characterize its taxonomic position. The genome sequence of *Isoptericola* sp. AK164 revealed the capacity of this strain as a PGPR, which could have potential use in agricultural and biotechnological applications. The combination of the present genomic data with comparative studies on gene expression and metabolite production in AK164 will deepen our understanding of which specific genes and pathways are induced during the plant bacterial interaction.

## Supplementary Information

Below is the link to the electronic supplementary material.

Supplementary file1 (DOCX 689 KB)

Supplementary file2 (XLSX 143 KB)
