# Supplementary material for: Complete genome sequence analysis of plant growth-promoting bacterium, Isoptericola sp. AK164 isolated from the rhizosphere of Avicennia marina growing at the Red Sea coast
Source: Arch Microbiol. 2023 Aug 14;205(9):307. doi: 10.1007/s00203-023-03654-1 (PMC10425560; doi:10.1007/s00203-023-03654-1)
Supplement: Supplementary file 1 — Supplementary file1 (DOCX 689 KB) [file 203_2023_3654_MOESM1_ESM.docx]

**Figure S1. BlastKOALA functional annotation of AK164 strain. The colored represent the number of genes assigned to each functional pathway.**

**
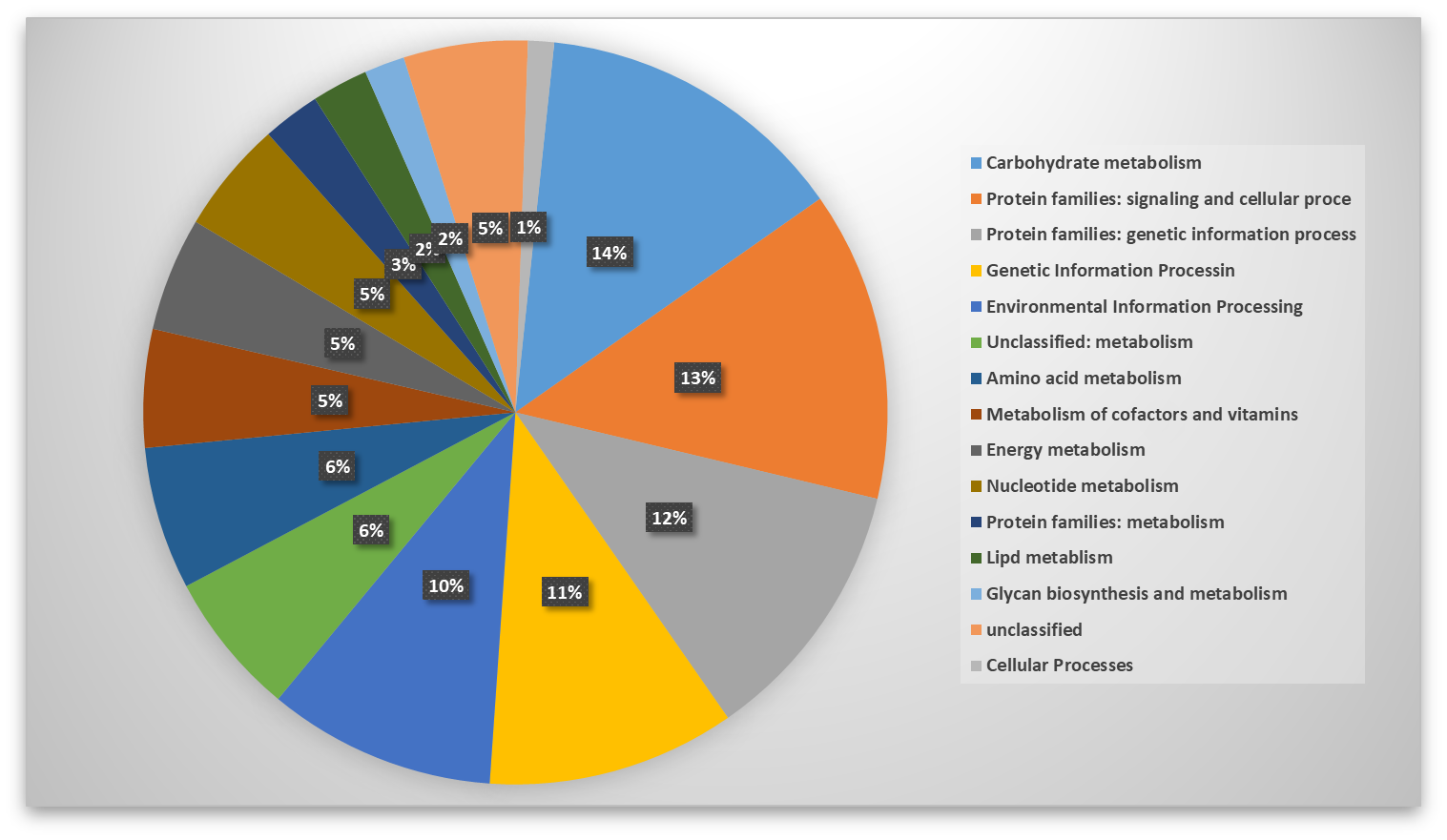
**

**Figure S2. KEGG functional annotation of AK164 strain. The colored bars represent the number of genes assigned to each functional pathway.**


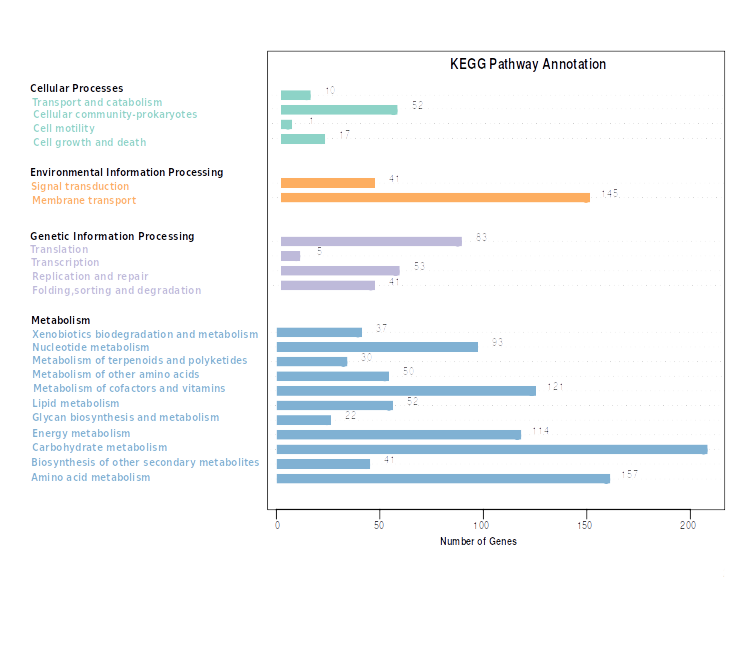


**Figure S3. GO functional annotation of AK164 strain. Colored bars represents the Percent and Number of genes assigned to each GO category.**
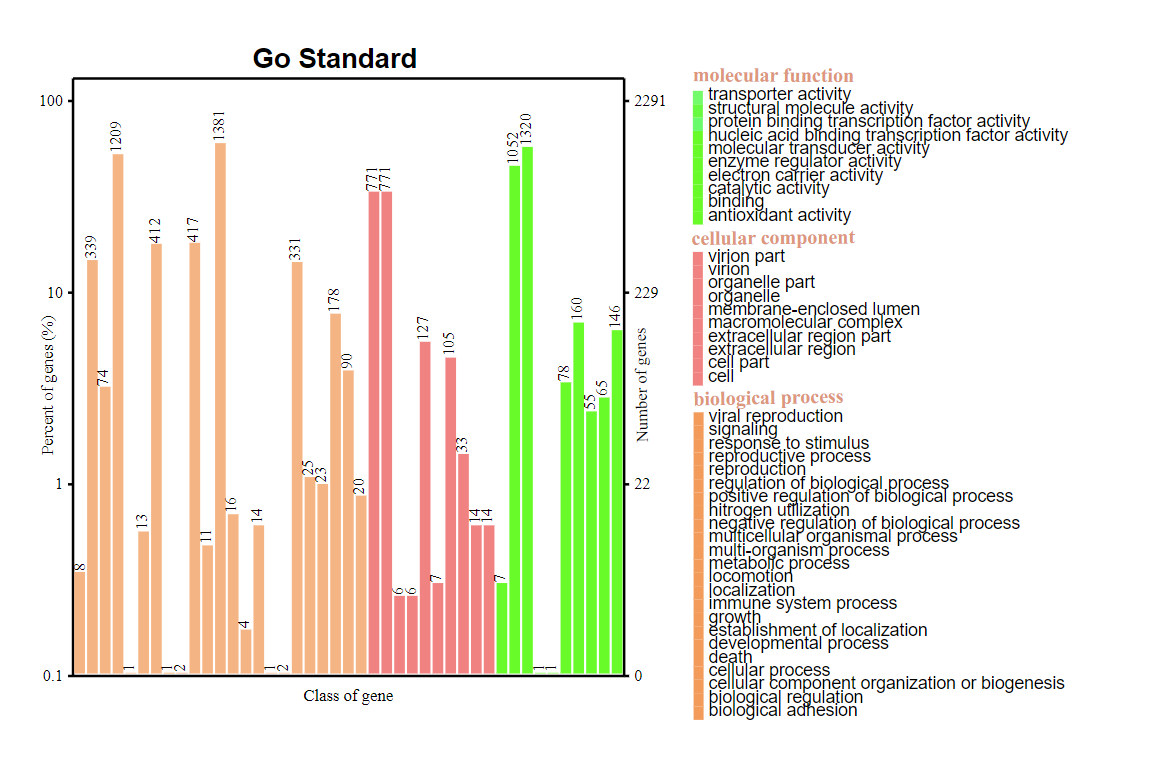


**Figure S4: Metabolic map of AK164 with highlight on Arginine and Proline metabolism**

**
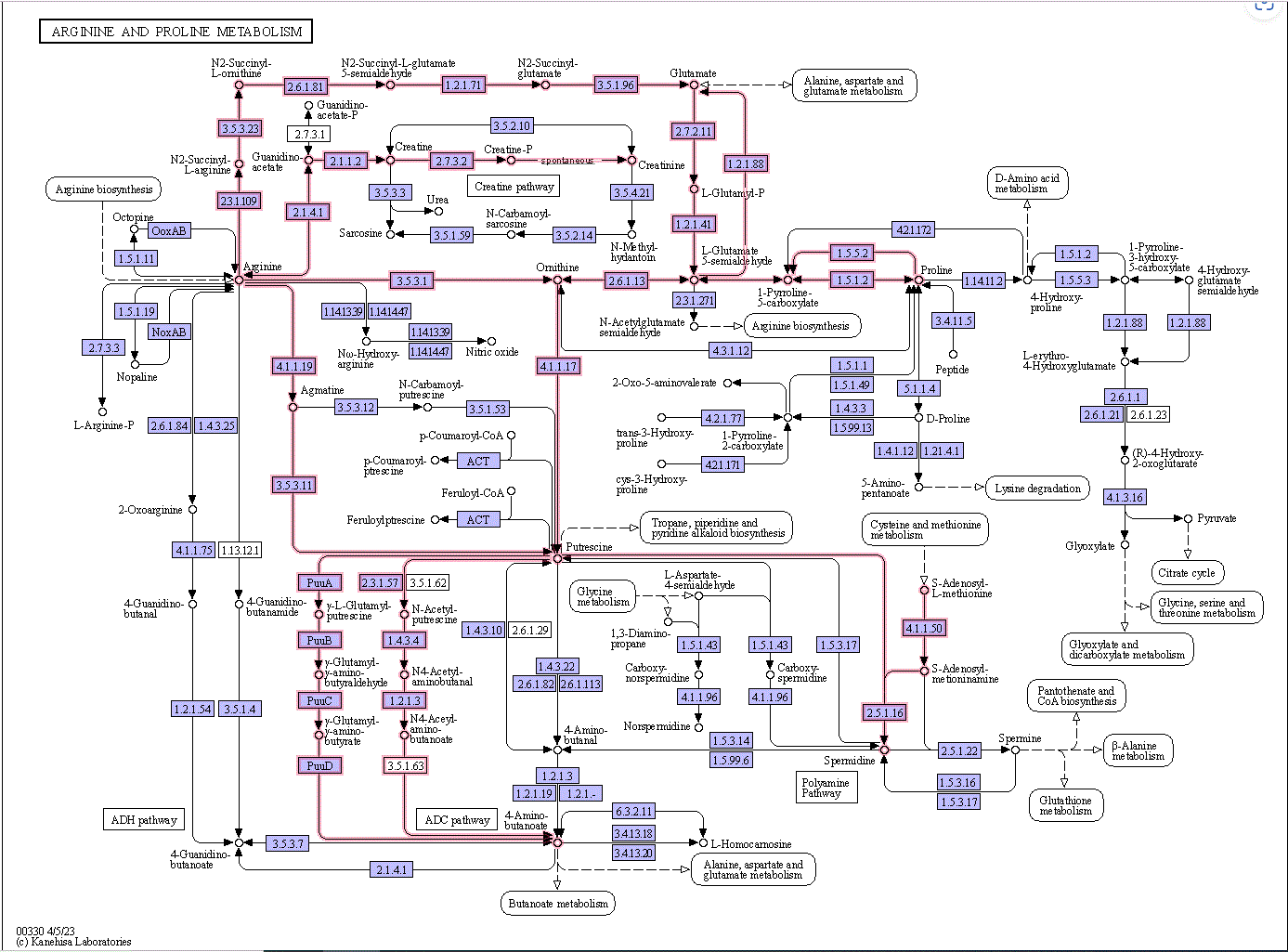
**
